# JMJD2A contributes to breast cancer progression through transcriptional repression of the tumor suppressor ARHI

**DOI:** 10.1186/bcr3667

**Published:** 2014-05-30

**Authors:** Li-Liang Li, Ai-Min Xue, Bei-Xu Li, Yi-Wen Shen, Yu-Hua Li, Cheng-Liang Luo, Ming-Chang Zhang, Jie-Qing Jiang, Zu-De Xu, Jian-Hui Xie, Zi-Qin Zhao

**Affiliations:** 1 Department of Forensic Medicine, School of Basic Medical Sciences, Fudan University, 138 Yixueyuan Road, Xuhui district, Shanghai 200032, P. R. China; 2 Department of Pathology, School of Basic Medical Sciences, Fudan University, 138 Yixueyuan Road, Xuhui district, Shanghai 200032, P. R. China

## Abstract

**Introduction:**

Breast cancer is a worldwide health problem and the leading cause of cancer death among females. We previously identified Jumonji domain containing 2A (JMJD2A) as a critical mediator of breast cancer proliferation, migration and invasion. We now report that JMJD2A could promote breast cancer progression through transcriptional repression of the tumor suppressor aplasia Ras homolog member I (ARHI).

**Methods:**

Immunohistochemistry was performed to examine protein expressions in 155 cases of breast cancer and 30 non-neoplastic tissues. Spearman correlation analysis was used to analyze the correlation between JMJD2A expression and clinical parameters as well as several tumor regulators in 155 cases of breast cancer. Gene and protein expressions were monitored by quantitative polymerase chain reaction (qPCR) and Western blot. Results from knockdown of JMJD2A, overexpression of JMJD2A, Co-immunoprecipitation (Co-IP) assay, dual luciferase reporter gene assay and chromatin immunoprecipitation (ChIP) elucidated molecular mechanisms of JMJD2A action in breast cancer progression. Furthermore, the effects of ARHI overexpression on JMJD2A-mediated tumor progression were investigated *in vitro* and *in vivo*. For *in vitro* experiments, cell proliferation, wound-healing, migration and invasion were monitored by cell counting, scratch and Boyden Chamber assays. For *in vivo* experiments, control cells and cells stably expressing JMJD2A alone or together with ARHI were inoculated into mammary fat pads of mice. Tumor volume, tumor weight and metastatic nodules were measured by caliper, electronic balance and nodule counting, respectively.

**Results:**

JMJD2A was highly expressed in human breast cancers and positively correlated with tumor progression. Knockdown of JMJD2A increased ARHI expression whereas overexpression of JMJD2A decreased ARHI expression at both protein and mRNA levels. Furthermore, E2Fs and histone deacetylases were involved in the transcriptional repression of ARHI expression by JMJD2A. And the aggressive behavior of JMJD2A in breast cancers could be reversed by re-expression of ARHI *in vitro* and *in vivo*.

**Conclusion:**

We demonstrated a cancer-promoting effect of JMJD2A and defined a novel molecular pathway contributing to JMJD2A-mediated breast cancer progression.

## Introduction

Breast cancer is a worldwide health problem threatening females. According to GLOBOCAN 2008 statistics, Breast cancer is the most frequently diagnosed cancer and the leading cause of cancer death among females, accounting for 23% of the total cancer cases and 14% of the cancer deaths [1]. Multiple factors, including genetic background, hormone disorder, and environmental impact are involved in breast cancer pathogenesis [2]. Current evidence shows that various genes contribute to breast cancer biological behavior and clinical phenotypes. It is reported that breast tumorigenesis is strongly associated with aberrant function of genes such as *HER-2/neu*, *BRCA1* and *CyclinD1*[3], which could be manipulated by gene expression level and/or gene mutation/rearrangement. In fact, dysregulation of gene expression, such as activation of oncogenes or inactivation of tumor-suppressor genes, are frequently reported to trigger breast cancers [4]. Genomics also elucidates that multiple gene mutations/rearrangements exist in breast cancers [5,6]. Therefore, multiple genes are involved in breast tumorigenesis. Further illustration of breast cancer pathogenesis is critical for disease treatment and prevention.

Recent discoveries of histone demethylases have advanced our understanding of transcriptional regulation [7-9]. Histone demethylases are enzymes that catalyze demethylation of lysine residues (mainly H3K4, H3K9, H3K27, H3K36 and H4K20) located in the N-terminal tails of histones. Based on recent findings, methylation of H3K9, H3K27 and H4K20 is mainly associated with repressive transcription whereas methylation of H3K4 and H3K36 mainly activates transcription [10]. Thus demethylation of different lysine residues may result in activated or repressed transcription. Jumonji domain containing 2A (JMJD2A, also known as JHDM3 or KDM4A) is a member of JmjC domain containing family JMJD2 that catalyzes histone demethylation. Due to its activity to demethylate di-and tri-methylation on a variety of histone lysine residues, such as H3K9 and H3K36 [11], H3K4 and H4K20 [12,13], JMJD2A can modify chromatin structure and function as a transcriptional repressor or activator. Previous report indicated that JMJD2A significantly demethylates tri- and di-methylated, but not monomethylated H3K36 and H3K9 *in vivo*[14]. Recent evidence shows that JMJD2A positively regulates the expression of *ADAM12*, *CXCL5* and *JAG1* genes through histone H3K9me3 demethylation [15]. Furthermore, it was observed that H3K9me3 levels are increased at *ASCL2* and *CHD5* gene promoters after depletion of JMJD2A [16,17]. JMJD2A is widely expressed in diverse cancers, including lung carcinoma, colon cancer and breast cancer [17-20]. In addition to its enzymatic activity, JMJD2A protein contains both leukemia-associated protein/plant homeodomain (LAP/PHD) and Tudor domains which were implicated protein-protein interactions. Functionally, JMJD2A could interact with histone deacetylase (HDAC) and retinoblastoma protein (pRb) and could direct repression of E2F-responsive promoters [21]. JMJD2A is also reported to be a novel N-CoR interacting protein, leading to transcriptional repression of downstream genes like *ASCL*2 [16].

Aplasia Ras homolog member I (ARHI) is a Ras-related small G-protein with a low guanosine triphosphate (GTP) enzymatic activity and Mg^2+^-dependence [22,23]. Unlike other small GTP-binding proteins, ARHI exhibits functional repression of cell growth and functions as a tumor suppressor. ARHI is highly expressed in normal breast and ovarian tissues, but repressed in breast and ovarian cancers [24,25], indicating that ARHI dysfunction is closely related with tumorigenesis and progression. In fact, overexpression of ARHI leads to retarded proliferation [26,27], migration [28], and invasion [27,28] in breast cancer. ARHI could restrict migration of non-cancer cells through interaction with C-RAF to suppress the activating phosphorylations on mitogen-activated protein kinase kinases (MEK) and extracellular signal-regulated kinase (ERK). And knockdown of ARHI could reverse the effect [29]. ARHI could also suppress ovarian cancer cell migration through inhibition of the Stat3 and FAK/Rho signaling pathways [30]. Like other tumor suppressors, ARHI expression could be regulated by deletion of an allele and promoter methylation [31], transcriptional factors and HDAC-containing complexes [32,33]. E2F1 and E2F4 are reported to negatively regulate ARHI expression by forming complex with HDAC. Overexpression of E2F1 and E2F4 could negatively regulate *ARHI* promoter activity [33], and multiple HDACs such as HDAC1, 3 and 11 are identified to negatively regulate *ARHI* expression [32].

Previously, we reported that knockdown of JMJD2A expression could slow down cell proliferation, migration and invasion in both MCF-7 and MDA-MB-231 cells [34,35]. However, the regulatory mechanisms remain unclear. In addition to *ASCL2* gene, JMJD2A was shown to transcriptionally repress other genes, such as the tumor suppressor gene *CHD5* in a lung carcinoma model [17]. In this study, we report that JMJD2A promotes breast cancer progression through transcriptional repression of the tumor suppressor ARHI. We found that JMJD2A correlates with breast cancer progression and promotes breast cancer progression through transcriptional silencing of *ARHI*. The repression of ARHI expression by JMJD2A required the involvements of E2Fs and HDACs, and the aggressive behavior of JMJD2A could be reversed by re-expression of ARHI both *in vitro* and *in vivo*. In all, we defined a molecular pathway contributing to JMJD2A-mediated breast cancer progression.

## Materials and methods

### Ethics statements

Permission to use human tissue sections for research purposes was obtained and approved by an institutional review board at Huashan Hospital, Shanghai, China. All patients provided their full consent to participate in our study. For animal research, the protocol was approved by the Ethics Committee from Shanghai Medical College, Fudan University, China. And all efforts were made to minimize suffering.

### Cells and reagents

Breast cancer cell lines MCF-7, T47D, SUM1315 and MDA-MB-231 were purchased from ATCC (Manassas, VA, USA) and cultured in DMEM (Hyclone, Logan, Utah, USA) supplemented with 10% FBS (Gibco, Los Angeles, CA, USA). Primary antibodies against JMJD2A (C37E5), E2F1 (3742), HDAC1 (5356) and HDAC3 (3949) were purchased from Cell Signaling Technology (Boston, MA, USA). Anti-estrogen receptor alpha (anti-ERα) (ab2746), anti-progesterone receptor (PR) (ab32085), anti-human epidermal growth factor receptor-2 (anti-HER2) (ab134182), anti-E2F4 (ab150360) and anti-ARHI (ab107051) primary antibodies were all obtained from Abcam (Cambridge, UK). Glyceraldehyde-3-phosphate dehydrogenase (GAPDH) antibody (sc-25778) was purchased from Santa Cruz (CA, USA). Lipofectamine 2000 was purchased from Invitrogen (Carlsbad, CA, USA). Trichostatin A (TSA) was purchased from Sigma Co. (St Louis, MO, USA). The chromatin immunoprecipitation (ChIP) kit was purchased from Upstate (a part of Millipore, Billerica, MA, USA). For the co-immunoprecipitation (Co-IP) assay, the protein G agarose beads and NP-40 lysis buffer were purchased from Beyotime Institute of Biotechnology (Nantong, China). Matrigel was purchased from BD Biosciences (San Jose, CA, USA). Cell counting kit-8 (cck-8) was purchased from Dojindo (Japan). And dual-luciferase reporter gene assay were from Promega (Madison, WI, USA).

### Plasmids and siRNAs

JMJD2A expression plasmid was subscribed by Dr Ralf Janknecht from the Department of Biochemistry and Molecular Biology, Mayo Clinic College of Medicine and Dr Hsing-Jien Kung, Deputy Director and Director of Basic Science, UC Davis Cancer Center. JMJD2A (NM_014663.2) was PCR-amplified from the subscribed plasmids and cloned into pcDNA3.1 vetors (pcDNA3.1-JMJD2A). ARHI (AY890085.1) was amplified on mRNA from MCF-7 cells and cloned into pcDNA3.1 vetors (pcDNA3.1-ARHI) as per the molecular cloning manual. siRNA specific to JMJD2A was chemically synthesized by Qiagen Technology Co. Ltd (Valencia, CA, USA). siRNA was diluted to 20 μmol/L with RNase-free water. siRNA duplexes were synthesized as follows: sense: 5′-GAGUUAUCAACUCAAGAUA-3′, antisense:5′-UAUCUUGAGUUGAUAACUC-3′. The other two JMJD2A siRNAs were synthesized by GenePharma (Shanghai, China) with the sequences described previously [15]. siRNAs specific to E2F1 and E2F4 were also synthesized by GenePharma (Shanghai, China) with sequences verified and described previously [36,37]. Scramble siRNA was used as negative control (NC) and sequences were as follows: sense: UUCUCCGAACGUGUCACGU, antisense: ACGUGACACGUUCGGAGAATT. Plasmids and siRNAs were transfected with Lipofectamine 2000 in accordance with the manufacturer’s instructions.

### Patients and histological and immunohistochemical (IHC) staining

All the 155 cases of breast cancer and 30 cases of non-neoplastic tissues were retrieved from the Department of Pathology, Huashan Hospital, Shanghai, China, within 2012. All cases were diagnosed by two experienced pathologists without discrepancy. None of the patients had received chemotherapy or radiation therapy previously. The paraffin-embedded tissues were first stained with hematoxylin and eosin (HE) for histological examination. Subsequently, sections were subjected to antigen retrieval by heating the slides in a microwave at 100°C for 10 minutes in 0.1-M citric acid buffer (PH 6.0), and then incubated with corresponding antibodies at 4°C overnight. After secondary antibody incubation at room temperature for 1 h, the slides were developed in 0.05% diaminobenzidine containing 0.01% hydrogen peroxidase. For negative controls, specific antibodies were replaced with normal goat serum by co-incubation at 4°C overnight preceding the immunohistochemiscal staining procedure.

### Western blot analysis

At 48 h after transfection, cells in different treatment groups were harvested. The procedure was used as described previously [38,39]. Antibodies against JMJD2A, ARHI, GAPDH, E2F1, and E2F4 were used to incubate target proteins overnight at 4°C. After the overnight incubation with the primary antibodies, membranes were washed and incubated with horseradish peroxidase (HRP)-labeled secondary antibody in Tris-buffered saline with Tween 20 (TBST) for 1 h. Immunoreactivity was detected with enhanced chemoluminescent autoradiography (ECL kit, Amersham, Pittsburgh, PA, USA), according to the manufacturer’s instruction. GAPDH was used as a loading control.

### Quantitative real-time PCR (qPCR)

Total RNA of each group were extracted respectively using Trizol solution (Invitrogen) at 24 h after transfection. Fast-strand cDNA was generated from 1 μg of total RNA using the PrimeScript RT Master Mix Perfect Real Time (TaKaRa, Shiga, Japan). Real-time qPCR was performed in an ABI PRISM 7500 Real-Time System. A 10-fold dilution of each cDNA was amplified in a 50 μl volume, using the SYBR Premix Ex TaqTM Perfect Real Time (TaKaRa, Shiga, Japan). The primers used were as follows: JMJD2A: forward 5′-ATCCCAGTGCTAGGATAATGACC-3′, reverse 5′-ACTCTTTTGGAGGAACCCTTG-3′; ARHI: forward 5′-GATTACCGCGTCGTGGTAGTC-3′, reverse 5′-TCAATGGTCGGCAGGTACTCA-3′; GAPDH: forward, 5′-TGACGCTGGGGCTGGCATTG-3′, reverse 5′-GCTCTTGCTGGGGCTGGTGG-3′.

Primers were synthesized by Shanghai Daweike Biotechnology Co. Ltd (Shanghai, China). PCR cycle conditions were 95°C for 30 s, and 40 cycles of 95°C for 5 s and 60°C for 34 s. The amplification specificity was evaluated with melting curve analysis. Relative mRNA was determined by using the formula 2^-ΔCT^ (CT, cycle threshold) where, as described previously [40]:

$$\mathrm{\Delta CT}=\mathrm{CT}\;\left(\mathrm{target}\;\mathrm{gene}\right)-\mathrm{CT}\;\left(\mathrm{GAPDH}\right).$$

### Dual luciferase reporter assay

HEK293T cells were seeded at a density of 2 × 10^5^/well in 24-well plates and co-transfected with indicated amounts of ARHI/luciferase reporter together with the full-length JMJD2A construct (JMJD2A-FL), the construct of JMJD2A with the substitution of histidine 188 by alanine (JMJD2A-H188A), or another construct of JMJD2A with the deletion of the tudor domains (JMJD2A-M867) [21,41]. Renilla luciferase plasmid (pRL) was co-transfected as internal control. Thirty-six hours after transfection, cells were lysed using passive lysis buffer and assayed immediately for reporter and control gene activities with the dual-luciferase reporter gene assay using a Lumat LB 9507 luminometer (EG & G Berthold, Bad Wildbad, Germany). To determine the effect of HDACs, cells were treated with or without 100 nM trichostatin A (TSA) at 12 h after transfection. Each experiment was performed in triplicate, and the data represent three independent experiments after normalization to renilla activity.

### Chromatin immunoprecipitation (ChIP) assay

Briefly, lysates were incubated with 4 μg of anti-JMJD2A antibody or normal rabbit IgG as a negative control. PCR amplification was performed using 1:100 dilution of input, an IgG negatively immunoprecipitated DNA and specific JMJD2A immunoprecipitated DNA. In general, samples were heated at 95°C for 3 minutes, followed by 31 cycles of 95°C for 30 s, 54°C for 30 s and 72°C for 20 s. After cycling, samples were incubated at 72°C for 10 minutes to permit completion of primers extension. Then PCR products were electrophoresed on a 2% agarose gel with ethidium bromide. The two pairs of *ARHI* primers (A1, A2) were as follows: A1 (−181 to 91) forward: 5′-TCGATTGTTGTAGATGCCAAG-3′, reverse: 5′-AGACTTACCTTTCTCGGAGGC-3′; A2 (−524 to −341), forward: 5′-TTTACCGGTCTTGCCACTAATG-3′, reverse: 5′-TCCAAAAGCAGTTTAATGCAGG-3′. *GAPDH* was used as a loading control (154 bp).

### Co-immunoprecipitation (Co-IP) assay

MDA-MB-231 cell lysates were obtained using NP-40 lysis buffer. Specific antibodies were used for immunoprecipitation as well as 20 μl of protein G agarose beads. The beads were washed in lysis buffer and boiled in 30 μl of SDS loading buffer; the entire sample was loaded on a SDS-polyacrylamide gel and processed by western blot. The membranes were immunoblotted with corresponding primary antibodies. Rabbit normal IgG was used as negative control.

### CCK-8 proliferation assay

Cells were seeded on 96-well plates at an initial density of 4 × 10^3^/well. At each monitored time point, cells of each well were stained with 10 μl CCK-8 (Dojindo, Japan) for 4 h at 37°C. Absorbance was measured using a synergy 2 multi-mode microplate reader (Bio Tek Instruments, Winooski, VT, USA) at 450 nm. All experiments were carried out in triplicate.

### Wound-healing assay and Boyden chamber assay

A wound-healing assay and Boyden chamber assay were performed as described previously [42]. Cells were plated on 6-well plates to form a confluent monolayer. Wounds made with sterile pipette tips were observed per 12 h. A migration assay was carried out using Boyden chambers (tissue culture-treated, 6.5-mm diameter, 8-μm pores, Transwell, Costar, Cambridge, MA, USA) containing polycarbonate membrane. For the invasion assay, 50 μl matrigel (BD Biosciences, San Jose, CA, USA) was used to mimic basement membrane. Briefly, 100 μl of 1 × 10^6^ cells in serum-free medium was added to the upper chamber and 600 μl of appropriate medium with 10% FBS was added to the lower chamber. Cells were incubated for 12 h. Migration cells on the under-surface of the membrane were fixed and stained with Giemsa for 10 minutes at room temperature. Photographs of five random regions were taken and the number of cells was counted to calculate the average number of migrated cells per plate.

### Mouse xenograft breast cancer models

Five-week-old female athymic nude mice (BALB/c^nu/nu^) were used for the experiment. Cells stably expressing JMJD2A or both JMJD2A and ARHI were constructed as previously described [43]. Cells (1 × 10^6^) were injected subcutaneously into the mammary fat pad of the mice. Mice were randomized (n = 7 per group) and assigned to specific groups. Tumor diameters were measured twice a week and tumor volumes (TV) were calculated using the formula as described [44]:

$$\mathrm{TV}=\left(L\times W^{2}\right)/2.$$

On day 27 after tumor cell injection, the mice were sacrificed and the excised tumors were measured and weighed. Lung and liver metastatic nodules were also calculated.

### Statistical analysis

All values are expressed as mean ± SD. The Student’s *t*-test and Spearman correlation analysis were used to evaluate the experimental data. *P* <0.05 was considered statistically significant.

## Results

### JMJD2A is highly expressed in breast cancer tissues

**Figure 1:**
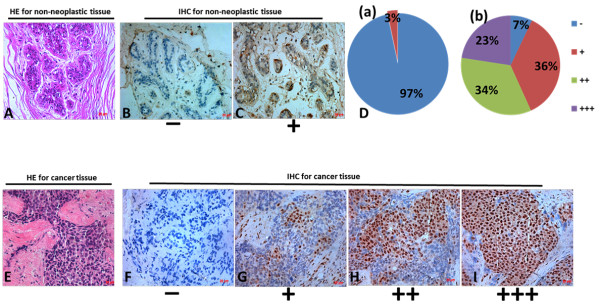
**Immunohistochemistry (IHC) analysis of JMJD2A expression in non-neoplastic and breast cancer tissues. (A)** Hematoxylin-eosin (HE) staining of non-neoplastic tissues. **(B, C)** Representative results of the negative staining **(B)** and weakly positive staining **(C)** of JMJD2A protein in non-neoplastic tissues. **(D)** Distributions of JMJD2A staining grades (−, +, ++ and +++) in both non-neoplastic tissues **(D, a)** and breast cancer tissues **(D, b)**. In non-neoplastic tissues, 29 out of 30 tissues exhibited to be JMJD2A negative and only 1 in 30 tissues exhibited to be JMJD2A weakly positive. In breast cancer tissues, the positive rate of JMJD2A staining in breast cancer tissues (93%) was significantly higher than that in non-neoplastic tissues (3%) (*P* <0.001). **(E)** Validation of breast cancer tissues by HE stain. **(F, G, H, I)** Representative results of negative staining (−, **F**), weakly positive staining (+, **G**), moderate staining (++, **H**) and strong staining (+++. **I**) of JMJD2A protein in breast cancer tissues are shown respectively. Scale bar = 20 μm.

**Figure 2:**
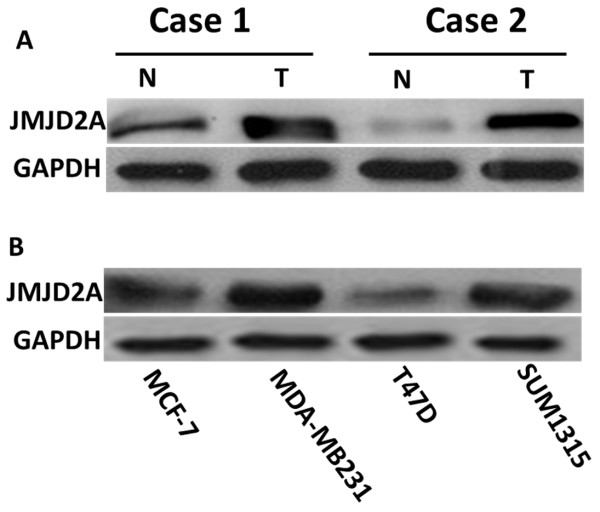
**Expression level of JMJD2A in breast cancer tissues and cell lines. (A)** Western blot analysis of JMJD2A protein from two random human primary breast cancer (T) and paired tumor-adjacent non-cancerous breast tissues (N), with each pair taken from the same patient. **(B)** Expression of JMJD2A protein was detected in breast cancer cell lines. Extracts from two low-metastatic breast cancer cell lines (MCF-7, T47D) and two highly metastatic breast cancer cell lines (SUM1315 and MDA-MB-231) were subjected to western blot analysis.

To investigate the role of JMJD2A in breast cancer, we examined JMJD2A expression in 155 human breast cancer tissues and 30 non-neoplastic breast tissues. Histological examination was first carried out on non-neoplastic breast tissues and breast cancer tissues, respectively (Figure 1A and E). Then all the tissues were analyzed by immunohistochemistry using anti-JMJD2A antibody and graded based on staining intensity (Figure 1B,C,F,G,H and I). We found that JMJD2A protein mainly localized in the nuclei and the positive rate in breast cancer tissues (93%, 144/155, Figure 1D (b)) was significantly higher than that in non-neoplastic tissues (3%, 1/30, Figure 1D (a)) (*P* <0.001). Among 30 non-neoplastic tissues, only one sample (3%) presented as weakly positive for JMJD2A, whereas the other 29 samples (97%) were all JMJD2A-negative. In contrast, 11 (7%) out of 155 cases of breast cancer tissues were negative, 56 (36%) were weakly positive, 53 (34%) were moderately positive and 35 (23%) were strongly positive. These data strongly support that JMJD2A expression is significantly increased in breast cancer tissues. Additionally, we randomly selected two primary breast cancer tissues and performed western blot analysis. This revealed that breast cancer tissues displayed higher JMJD2A expression level than that in paired tumor-adjacent non-cancerous tissues in the two samples (Figure 2A). These results suggest that JMJD2A is highly expressed in breast cancer.

### Expression of JMJD2A is positively correlated with progression of breast cancer and negatively with tumor suppressor ARHI

To determine whether the expression level of JMJD2A was associated with progression of breast cancer, we initially examined JMJD2A expression in four breast cancer cell lines, including two weakly metastatic cell lines (MCF-7 and T47D) and two highly metastatic cell lines (MDA-MB-231 and SUM1315). Compared with the weakly metastatic cell lines, highly metastatic cell lines had a higher expression level of JMJD2A (Figure 2B). We then carried out Spearman correlation analysis to determine the relationship between JMJD2A expression and clinical parameters (Table 1). Statistical analysis revealed that there was a significantly positive correlation of JMJD2A expression with tumor, node, metastasis (TNM) stage (*P* = 0.004, *r* = 0.227), and tumor size (*P* = 0.026, *r* = 0.179), but JMJD2A expression did not significantly correlate with lymph node metastasis (*P* = 0.102), or with age (*P* = 0.092). These results reveal that JMJD2A positively relates to tumor progression.

**Table 1:** Association between JMJD2A expression and disease parameters in 155 cases of breast cancer

	**Total**	**JMJD2A**				***P*-value**	**Correlation coefficient**
		**0 (−)**	**1 (+)**	**2 (++)**	**3 (+++)**		
		**n = 11**	**n = 56**	**n = 53**	**n = 35**		
TNM^1^ stage							
I and II	139	10	54	49	26	0.004**	0.227
III and IV	16	1	2	4	9		
Tumor size							
≤2 cm	110	6	48	37	19	0.026*	0.179
2 cm < T ≤5 cm	42	5	7	14	16		
>5 cm	3	0	1	2	0		
Lymph node metastasis							
Negative	100	9	29	35	27	0.102	−0.132
Positive	55	2	27	18	8		
Age							
<50 years	46	4	21	13	8	0.092	0.136
≥50 years	109	7	35	40	27		

^1^TNM stage is a classification of malignant tumors used by the International Federation of Gynecology and Obstetrics (FIGO). T describes the size of primary tumor, N describes nearby lymph nodes that are involved, and M describes distant metastasis. *P <0.05, **P <0.01.

**Figure 3:**
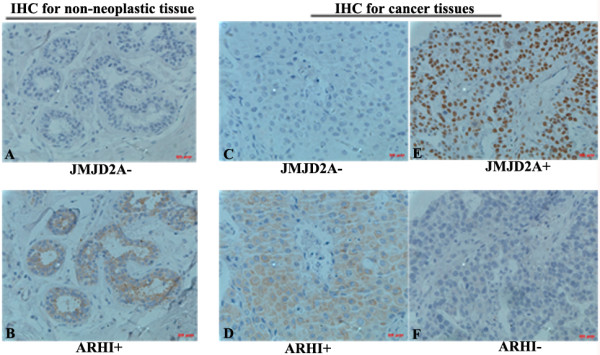
**JMJD2A is negatively correlated with Aplasia Ras homolog member I (ARHI) in breast tissues.** Serial sections were stained with JMJD2A and ARHI antibodies, respectively. **(A)** Representative result of negative JMJD2A staining in serial sections from non-neoplastic tissues. **(B)** Representative result of positive ARHI staining in serial sections from non-neoplastic tissues. **(C)** Representative result of negative JMJD2A staining in serial sections from breast cancer tissues. **(D)** Representative result of positive ARHI staining in serial sections from breast cancer tissues. **(E)** Representative result of positive JMJD2A staining in serial sections from breast cancer tissues. **(F)** Representative result of negative ARHI staining in serial sections from breast cancer tissues. Scale bar = 20 μm.

We subsequently analyzed the relationship of JMJD2A expression with tumor regulators including estrogen receptor (ER)α, progesterone receptor (PR), HER2 and ARHI. Our results showed that ERα and PR were stained mainly in the nuclei and HER2 was localized in the cell membrane (data not shown), whereas ARHI was mainly in the cell membrane and partially in the cytoplasm (Figure 3B and D). Spearman correlation analysis showed that expression of JMJD2A was inversely correlated with expression of ARHI (*P* = 0.016, *r* = −0.194, Table 2). However we did not observe a significant association between JMJD2A immunoreactivity and other tested clinicopathological parameters, including ERα (*P* = 0.778), PR (*P* = 0.147) and HER2 (*P* = 0.742). This result implies that JMJD2A may be negatively linked with the tumor suppressor ARHI.

**Table 2:** Association between JMJD2A expression and clinicopathological parameters in 155 cases of breast cancer

	**Total**	**JMJD2A**				***P*-value**	**Correlation coefficient**
		**0 (−)**	**1 (+)**	**2 (++)**	**3 (+++)**		
		**n = 11**	**n = 56**	**n = 53**	**n = 35**		
ERα							
Negative	52	4	18	17	13	0.778	−0.023
Positive	103	7	38	36	22		
PR							
Negative	77	7	22	26	22	0.147	−0.117
Positive	78	4	34	27	13		
HER2							
Negative	7	0	3	2	2	0.742	−0.027
Positive	148	11	53	51	33		
ARHI							
Negative	63	20	12	17	14	0.016*	−0.194
Positive	92	40	24	21	7		

*P <0.05. ER, estrogen receptor; PR, progesterone receptor; HER2, human epidermal growth factor receptor-2; ARHI, Aplasia Ras homolog member I.

To verify the negative relationship between JMJD2A and ARHI, we firstly performed western blot analysis using human breast cancer tissues and paired tumor-adjacent non-cancerous tissues. As expected, ARHI was downregulated in JMJD2A-overexpressed cancer tissues and upregulated in tumor-adjacent tissues (Additional file 1: Figure S1). We then carried out IHC staining on serial sections with both anti-JMJD2A and ARHI antibodies in non-neoplastic breast tissues and breast cancer tissues. Our results showed that JMJD2A-negative staining sections were ARHI-positive both in non-neoplastic tissues (Figure 3A and B) and cancer tissues (Figure 3C and D), whereas ARHI-negative staining sections were JMJD2A-positive in cancer tissues (Figure 3E and F). Taken together, JMJD2A expression inversely correlated with ARHI expression in both non-neoplastic tissues and breast cancer tissues.

### JMJD2A binds to *ARHI* promoter and negatively controls its promoter activity

**Figure 4:**
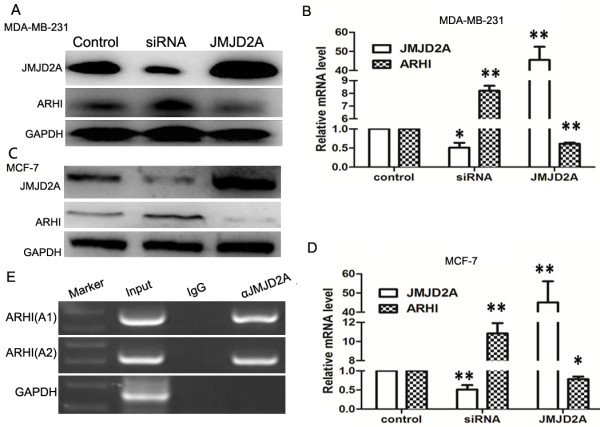
**JMJD2A binds to *ARHI *gene promoter and represses *ARHI *transcriptional activity. (A, B)** Western blot and quantitative real-time PCR (qPCR) analysis of expressions of JMJD2A and ARHI in breast cancer cell line MDA-MB-231. siRNA against JMJD2A and JMJD2A expression plasmid were used, respectively. ARHI expression level was upregulated in the siRNA group and downregulated in the JMJD2A group. **(C, D)** Western blot and qPCR analysis of expressions of JMJD2A and ARHI in breast cancer cell line MCF-7. The results were proved similar to those in breast cancer cell line MDA-MB-231. **(E)** JMJD2A binds to the *ARHI* gene promoter with two binding sites (A1 and A2). Anti-JMJD2A antibody was adopted. Normal IgG was used as negative control. The A1 site is critical for histone deacetylase (HDAC) binding with the *ARHI* promoter while A2 for E2Fs binding with *ARHI* promoter. *GAPDH* was used as a loading control. Two binding sites were both verified. *P <0.05, **P <0.01. ARHI, Aplasia Ras homolog member I; GAPDH, glyceraldehyde-3-phosphate dehydrogenase.

To further investigate the role of JMJD2A in ARHI expression, we carried out western blot analysis *in vitro* with weakly-metastatic cell line MCF-7 and highly metastatic cell line MDA-MB-231. siRNA specific against JMJD2A (siRNA group) and JMJD2A expression plasmid (pcDNA3.1-JMJD2A (JMJD2A)) were transfected into both cell lines. Transfection efficiency was approximately 71.3% (data not shown). Our results showed that siRNA-induced JMJD2A silencing caused a significant increase in ARHI expression whereas overexpression of JMJD2A led to a decrease in ARHI expression both in MDA-MB-231 cells (Figure 4A) and MCF-7 cells (Figure 4C). Two additional siRNAs against JMJD2A also elucidated the negative relationship between JMJD2A and ARHI expression at protein level (Additional file 2: Figure S2). Consistently, real-time qPCR analyses indicated that mRNA of *ARHI* was elevated in the siRNA group and reduced in the JMJD2A group (Figure 4B and D), suggesting that JMJD2A negatively regulated ARHI expression at transcriptional level. Recent evidence shows that JMJD2A could interact with HDACs and pRb [21], and E2F-HDAC complex functions as a transcriptional repressor of *ARHI*[33]. In fact, two binding sites of transcription factors at the *ARHI* promoter were reported including the site bound by E2Fs (from −524 to −341, indicated as the A2 site) and another site directly or indirectly bound by HDACs (from −181 to 91, indicated as the A1 site) [32,33]. Thus we hypothesized that JMJD2A might bind to the *ARHI* promoter by interaction with E2Fs (mainly E2F1 and E2F4) and HDACs (mainly HDAC1 and HDAC3). We first assessed whether JMJD2A could immunoprecipitate the *ARHI* promoter. DNA immunoprecipitated by antibodies was then amplified by PCR with two pairs of primers specific for the respective binding sites. As expected, no DNA fragment was detected when normal IgG was used. In contrast, DNA fragments immunoprecipitated by JMJD2A antibody yielded a 183-base product that spanned nucleotides (−524 to −341) (Figure 4E), suggesting that JMJD2A could directly or indirectly bind to the binding site of E2Fs. Moreover, we could also amplify the DNA fragment (−181 to 91) to observe a 270-base product (Figure 4E), suggesting that JMJD2A might form a complex with HDACs and/or other transcription factors. Therefore, JMJD2A could bind to the promoter of ARHI to inhibit the expression of ARHI.

### E2Fs and HDACs are involved in JMJD2A binding to the *ARHI* promoter

**Figure 5:**
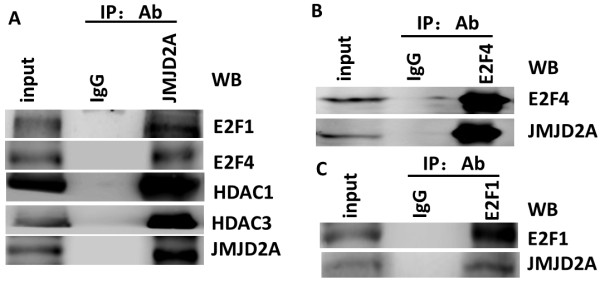
**Interactions between JMJD2A, E2F1, E2F4, histone deacetylase (HDAC)1 and HDAC3. (A)** JMJD2A could immunoprecipitate E2F1, E2F4, HDAC1 and HDAC3. **(B)** JMJD2A could be immunoprecipitated by E2F1 with anti-E2F1 antibody. **(C)** JMJD2A could be immunoprecipitated by E2F4 with anti-E2F4 antibody. IP, immunoprecipation; Ab, antibody.

**Figure 6:**
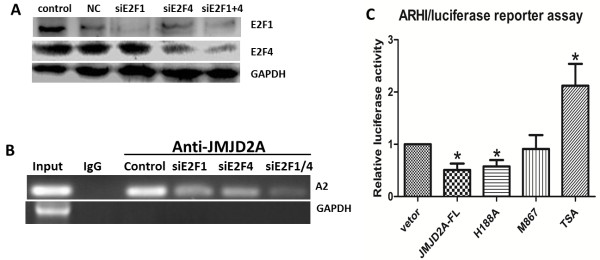
**E2F and histone deacetylase (HDAC) are involved in the transcriptional inhibition of ARHI by JMJD2A. (A)** Specific siRNAs against E2F1 and E2F4 were efficient to knockdown E2F1 and E2F4 expression level. **(B)** Effects of E2Fs knockdown on JMJD2A binding to the A2 site of the *ARHI* promoter. Both siRNAs could inhibit the binding of JMJD2A to the *ARHI* promoter. **(C)** Effects of JMJD2A-FL, JMJD2A mutants (H188A and M867) and trichostatin A (TSA) on *ARHI* promoter activity. *P <0.05, **P <0.01. ARHI, Aplasia Ras homolog member I.

To uncover the detailed mechanism of JMJD2A binding to the ARHI promoter, we first investigated the interactions of JMJD2A, E2Fs and HDACs. As shown in Figure 5A, JMJD2A specifically co-immunoprecipitated E2F1, E2F4, HDAC1 and HDAC3. In fact, HDAC1 and HDAC3 were previously reported to immunoprecipitate JMJD2A [21]. E2F1 and E2F4, respectively, also immunoprecipitated JMJD2A (Figure 5B and C), suggesting that E2Fs and HDACs could form a complex with JMJD2A. We then synthesized specific siRNAs against E2F1 and E2F4 to determine whether E2Fs are required for JMJD2A binding to the *ARHI* promoter. Two siRNAs were proved to work efficiently (Figure 6A). Subsequent ChIP assay showed that band density was lower when either siE2F1 or siE2F4 was transfected. And after co-transfection of both siRNAs, band density was further decreased (Figure 6B), suggesting that JMJD2A binding with the A2 site (−524 to −341) in the *ARHI* promoter required the involvements of E2Fs. Tudor domain of JMJD2A is critical for protein interaction and the JmjC domain is pivotal for enzymatic activity. We constructed two mutants of JMJD2A expression plasmid, namely JMJD2A-M867 and JMJD2A-H188A, respectively and performed a dual luciferase reporter assay. Our results showed that JMJD2A-H188A could inhibit the *ARHI* promoter activity as did by JMJD2A-FL whereas JMJD2A-M867 partially lost the inhibition effect (Figure 6C), indicating that the Tudor domains, but not the catalytic activity, of JMJD2A might be required for JMJD2A-mediated repression of promoter activity. Furthermore, TSA treatment could impair JMJD2A-mediated inhibition of the *ARHI* promoter activity, corroborating the conclusion that JMJD2A-mediated repression requires HDACs. Overall, JMJD2A binding to the *ARHI* promoter requires the involvement of E2Fs and HDACs.

### ARHI reverses JMJD2A-induced tumor progression *in vitro* and *in vivo*

**Figure 7:**
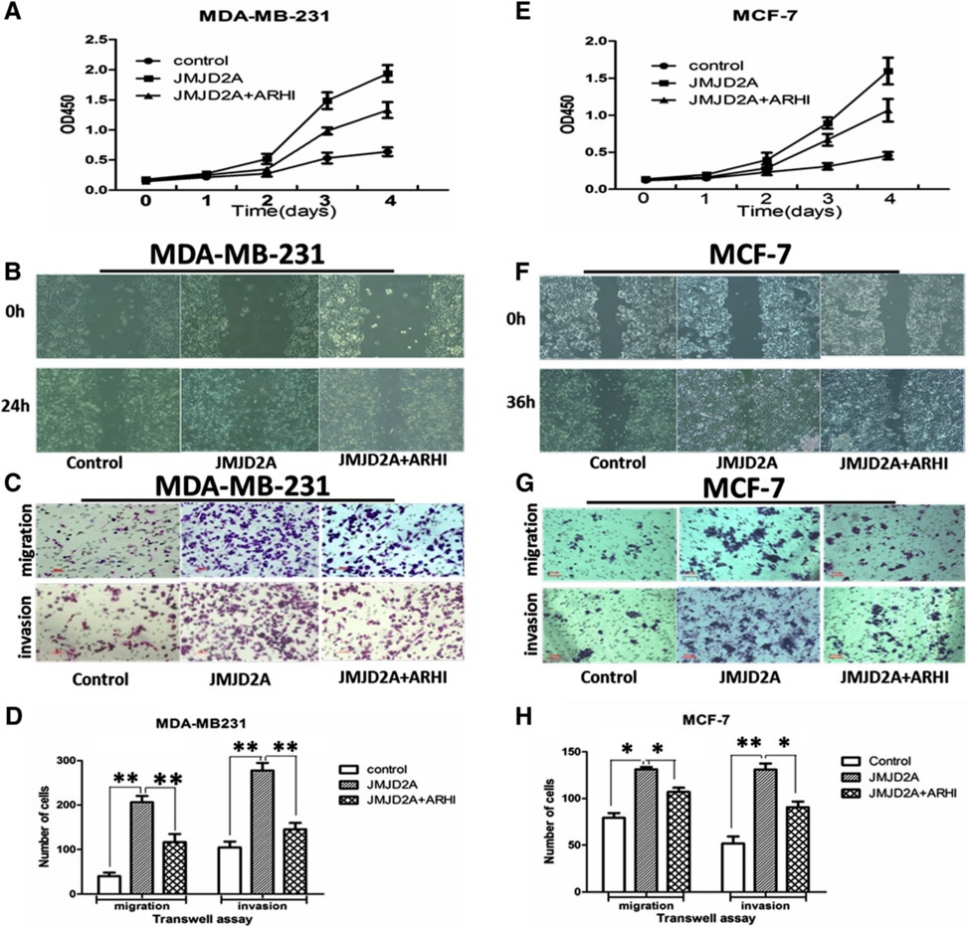
**ARHI reverses JMJD2A-induced tumor progression *in vitro.*** MDA-MB-231 cells and MCF-7 cells were transfected with empty vetor, pcDNA3.1-JMJD2A plasmid, and pcDNA3.1-JMJD2A&pcDNA3.1-ARHI plasmids in advance. **(A, E)** ARHI reversed JMJD2A-mediated cell proliferation by day 2 and inhibited JMJD2A-mediated proliferation by up to 40% in MDA-MB-231 cells **(A)** and 33% in MCF-7 cells **(E)** on day 4. **(B, F)** Two cell lines were analyzed using a wound-healing assay. Motility was compared between different groups. At 24 h ARHI slowed down JMJD2A-promoted motility in MDA-MB-231 cells **(B)**. At 36 h ARHI slowed down JMJD2A-promoted motility in MCF-7 cells **(F)**; magnification 200×. **(C, G)** A transwell assay was performed on MDA-MB-231 cells **(C)** and MCF-7 cells **(G)**. ARHI was observed to attenuate JMJD2A-promoted migration and invasion ability; scale bar = 20 μm. **(D, H)** The transwell assay was photographed and migrated/invaded cell numbers of five random regions were counted to calculate the average number of cells that transmigrated. *P <0.05, **P <0.01. ARHI, Aplasia Ras homolog member I.

To reinforce the link between ARHI and JMJD2A, we investigated the role of ARHI during JMJD2A overexpression-mediated tumor progression. To this end, the inhibitory effects of ARHI on cell proliferation, migration, and invasion were first confirmed *in vitro* and *in vivo* (Additional file 3: Figure S3). Next, cell proliferation was detected in MCF-7 cells and MDA-MB-231 cells transfected with JMJD2A alone or along with ARHI. We found that ARHI significantly suppressed JMJD2A-induced tumor cell hyper-proliferation (Figure 7A and E). The proliferative rate was dampened by approximately 40% in MDA-MB-231 cells and 33% in MCF-7 cells on day 4, when ARHI was co-transfected. The wound-healing assay also showed that JMJD2A-mediated motility was inhibited by overexpression of ARHI (Figure 7B and F). Similarly, compared with the JMJD2A group, both migration and invasion abilities were also reduced after co-transfection of JMJD2A and ARHI plasmids (Figure 7C and G). In the migration assay, ARHI reduced migratory cells by 43.5% in MDA-MB-231 cells (Figure 7D) and 18.5% in MCF-7 cells (Figure 7H) compared with the JMJD2A group. In the invasion assay, ARHI decreased invasive cells by 47.8% in MDA-MB-231 cells (Figure 7D) and 30.8% in MCF-7 cells (Figure 7H) compared with the JMJD2A group.

**Figure 8:**
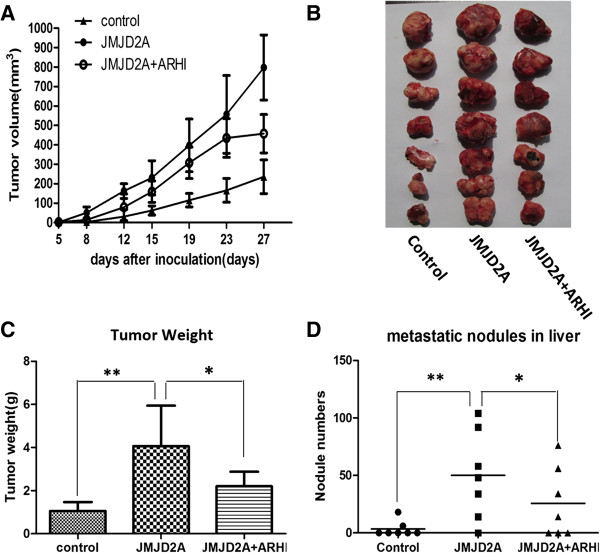
**ARHI reverses JMJD2A-induced tumor progression *in vivo *in a xenograft model.** MDA-MB-231 cells stably expressing JMJD2A alone or along with ARHI were constructed. Cells treated with empty vetor were used as control. **(A)** Periodic detection of tumor volume in three groups. **(B)** Tumors were dissected and each tumor in three groups was exhibited. **(C)** Tumors excised were weighed on day 27. **(D)** Breast cancer metastasis was observed and liver nodules were counted in three groups. Total nodule numbers were statistically analyzed. *P <0.05, **P <0.01. ARHI, Aplasia Ras homolog member I.

To further investigate the role of ARHI on JMJD2A-induced tumorigenesis *in vivo*, we used a female athymic mice model of breast cancer. Tumor diameters were measured regularly to calculate tumor volumes (Figure 8A). Volume measurement showed that tumors displayed a smaller size in volume in each monitored time point when ARHI was co-transfected (Figure 8A). Tumors were excised on day 27 and also weighed (Figure 8B and C). Consistently, compared with the average weight when JMJD2A was overexpressed alone, a lower mass in weight was also observed when ARHI was overexpressed together with JMJD2A (Figure 8B and C). Actually, compared with JMJD2A group, tumor growth was slowed down by nearly 50% on day 27 when ARHI expression plasmid was transfected along with JMJD2A expression plasmid (Figure 8C). During dissection, we observed metastatic nodules in lung and liver. Nodules in each group of mice were counted to calculate average nodule numbers. Our results showed that average metastatic nodules in liver in each group were significantly different (Figure 8D, *P <0.05 versus the JMJD2A group). This was in accordance with our results showing that ARHI suppressed JMJD2A overexpression-induced tumor migration and invasion *in vitro*. However, we did not observe a significant difference in the average number of metastatic nodules in lung (data not shown). In all, ARHI could invert JMJD2A-induced tumor progression.

## Discussion

JMJD2A is involved in diverse cancers, including lung carcinoma [17], colon cancer [19] and breast cancer [18,20]. We previously found that mRNA level of JMJD2A is negatively correlated to that of the tumor suppressor ARHI in breast cancer [20]. Here, we demonstrated a breast cancer-promoting effect of JMJD2A and the regulatory mechanism of ARHI expression by JMJD2A.

Recently, numerous HDAC-containing repression complexes have been identified [32,33,45-49]. Among them, E2F-HDAC repressor is characterized as an important one. Here we demonstrated that transcriptional repression of ARHI by JMJD2A requires the involvement of E2F and HDAC. ARHI was downregulated by JMJD2A at both protein and mRNA level. And *ARHI* promoter activity was significantly inhibited by JMJD2A, indicating a transcriptional repression of *ARHI* by JMJD2A. Current evidence shows that JMJD2A could interact with HDAC (mainly HDAC1 and HDAC3) and pRb [21]. E2F1 and E2F4 could mediate repression of *ARHI* promoter activity on the identified binding site (A2: −524 to −341) together with HDAC and/or pRb, respectively [33]. ChIP assay suggested that JMJD2A could immunoprecipitate the A2 site of the *ARHI* promoter (Figure 4E). Co-IP assay revealed the interactions of JMJD2A with E2F1, E2F4, HDAC1 and HDAC3 (Figure 5A). Furthermore, knockdown of E2F1 and/or E2F4 significantly suppressed the recruitment of JMJD2A to the A2 site in the *ARHI* promoter (Figure 6B). Therefore, JMJD2A could form a complex with E2Fs and HDACs to repress the *ARHI* promoter activity. Additionally, multiple HDACs (mainly HDAC1 and HDAC3) are also reported to directly or indirectly bind to the *ARHI* promoter at the site from −181 to 91 (A1) [32]. Inhibition of HDAC activity by TSA treatment significantly increased *ARHI* promoter activity (Figure 6C). However, as revealed by analysis of *ARHI* promoter sequence, only transcription factors like Sp1 and PEA3 were specifically predicted to bind *ARHI* promoter site from −181 to 91 [50]. HDAC could not specifically bind to the A1 site in the *ARHI* promoter. We speculate that there might be other transcription factors such as Sp1, PEA3 which forms a complex with JMJD2A and HDACs at the A1 site. Collectively, binding of JMJD2A to the *ARHI* promoter is E2F- and HDAC-dependent. Moreover, ARHI re-expression reversed JMJD2A-induced tumor progression *in vitro* (Figure 7) and *in vivo* (Figure 8), which in turn corroborated our results that JMJD2A promotes breast cancer progression through transcriptional repression of the tumor suppressor ARHI. Taken together, we defined a novel gene regulated by JMJD2A in breast cancer.

Intriguingly, ERα was not observed to significantly correlate with JMJD2A (*P* = 0.778). As a member of the same family, JMJD2B was reported to interact with ERα and SWI/SNF-B complex upon estrogen stimulation and lead to ERα target genes activation [51]. In this process, JmjC catalytical domain, a structure harbored by both JMJD2A and JMJD2B, is crucially required. Thus, JMJD2A was thought to participate in breast cancer onset through the ERα signaling pathway. However, the report further showed that depletion of JMJD2A caused only a marginal defect in ERα target gene induction, indicating that JMJD2A interaction with ERα was not robust. These results are in line with our findings.

The identification of *ARHI* as a gene regulated by JMJD2A is of biological significance. JMJD2A was reported to transcriptionally repress *ASCL*2 *in vitro*[16,17] and *CHD*5 in lung carcinoma [17]. Genes regulated by JMJD2A in breast cancer have not been reported. Our report may extend the list of downstream genes regulated by JMJD2A. Moreover, the tumor suppressor ARHI is frequently downregulated in breast cancers [22-25]. Here we further demonstrated the regulatory network contributing to ARHI inactivation in breast cancers.

The effect of chromatin structure on gene transcription is determined, at least in part, by the posttranscriptional modifications of the histones, such as acetylation and methylation [52]. Acetylation is believed to facilitate transcription whereas deacetylation reverses such effects and thus, reinforces the repressive effect of chromatin [52]. As histone deacetylase, HDAC possibly plays key roles in the repression of transcription. Our results showed that inhibition of ARHI promoter activity by JMJD2A required the involvement of HDAC. In fact, HDAC has functional links to histone acetylation and mediates *ARHI* repression [32]. In contrast, histone demethylation might not be required. Similar to JMJD2A-FL, JMJD2A-H188A could also repress *ARHI* promoter activity. Moreover, JMJD2A contains LAP/PHD and Tudor domains, which are implicated in protein-protein interactions [21]. In this way, JMJD2A could recruit co-regulators, such as pRb, E2F and HDAC. Actually, deletion of Tudor domains impaired JMJD2A-mediated repression of *ARHI* promoter activity (Figure 6C). It is possible that JMJD2A loses the ability to bind to promoters or to recruit the regulatory factors after deletion of the Tudor domains.

One interesting observation is the significant liver instead of lung metastasis in the xenograft model. Metastasis is common in malignant tumors. The liver represents a common site of metastasis for solid cancers and the third most common site for breast cancer metastasis [53]. The metastatic cascade consists of numerous steps, in which interactions between primary tumor cells and resident cells represent the most important factors determining to which organs tumors metastasize [54,55]. The importance of cancer cell-hepatocyte interactions was reinforced by the observation that colorectal cancer cells also interact with hepatocytes when metastasizing to liver [56,57]. As adhesion molecules such as claudin-2 are reported to promote breast cancer liver metastasis by facilitating tumor cell interactions with hepatocytes [58,59], we speculate that there must be other molecules regulated by JMJD2A that mediate breast cancer preferably liver metastasis. Further studies will be required to understand the mechanism.

Collectively, JMJD2A promotes breast cancer progression through transcriptional repression of the tumor suppressor ARHI. Of note, JMJD2A was predicted to be a transcriptional repressor and activator [17]. Here we elucidate JMJD2A as a transcriptional repressor of tumor suppressor ARHI. Genes transcriptionally activated by JMJD2A in breast cancer may also potentially exist. Other genes repressed/activated by JMJD2A in breast cancer remain to be exploited.

## Conclusion

In summary, our data indicate that JMJD2A could promote breast cancer progression through transcriptional repression of the tumor suppressor ARHI. In the clinic, JMJD2A associates with tumor progression. JMJD2A could also promote breast cancer cell proliferation, invasion and migration which could be reversed by ARHI re-expression. The repression of ARHI expression by JMJD2A requires the involvements of E2Fs and HDACs.

## Abbreviations

ARHI: Aplasia Ras homolog member I; ASCL2: Achaete scute-like homologue 2; bp: base pairs; CHD5: Chromodomain helicase DNA-binding protein 5; ChIP: chromatin immunoprecipitation; Co-IP: co-immunoprecipitation; CT: cycle threshold; DMEM: Dulbecco’s modified Eagle’s medium; ERα: Estrogen receptor alpha; FBS: fetal bovine serum; GAPDH: Glyceraldehyde-3-phosphate dehydrogenase; HDAC: Histone deacetylase; HE: hematoxylin and eosin; HER2: Human epidermal growth factor receptor-2; IHC: immunohistochemistry; JMJD2A: Jumoji domain containing 2A; PR: Progesterone receptor; qPCR: quantitative real-time PCR; TSA: trichostatin A.

## Competing interests

The authors declare that they have no competing interests.

## Authors’ contributions

LLL and AMX performed the western blot, qPCR, proliferation assay, wound-healing assay and Boyden chamber assay and drafted the whole manuscript. BXL and YWS contributed to the ChIP assay, Co-IP assay and statistical analysis. CLL and YHL carried out the *in vivo* studies. MCZ, JQJ and ZDX prepared tissue sections and participated in IHC analysis. JHX designed the whole study and carried out the dual luciferase reporter assay. ZQZ conceived of the study and revised the manuscript. All authors read and approved the final manuscript.

## Supplementary Material

Additional file 1: Figure S1 JMJD2A is negatively correlated with Aplasia Ras homolog member I (ARHI) expression in breast cancer tissues. Western blot analysis of both JMJD2A and ARHI expression levels in two random human primary breast cancer (T) and paired tumor-adjacent non-cancerous breast tissues (N), with each pair taken from the same patient. Click here for file

Additional file 2: Figure S2 Knockdown of JMJD2A could upregulate Aplasia Ras homolog member I (ARHI) expression. Two additional specific siRNAs against JMJD2A were synthesized and transfected, respectively. Negative control siRNA was also used. Both JMJD2A and ARHI expression were detected with western blot analysis. Glyceraldehyde-3-phosphate dehydrogenase (GAPDH) was used as loading control. Click here for file

Additional file 3: Figure S3 Aplasia Ras homolog member I (ARHI) inhibits the tumor progression *in vitro* and *in vivo*. MDA-MB-231 cells with (ARHI) or without (Control) stable expressions of ARHI were generated using the Lenti-X system (Clontech). Analysis of cell proliferation (**A**), migration (wound-healing assay, **B**) and invasion (**C**) were performed. (**D**) The *in vivo* effect of ARHI was evaluated in the mouse xenograft model and the tumor weight was calculated on day 25 (n = 6 for each group). *P <0.05. Click here for file
