# Erratum to: JMJD2A contributes to breast cancer progression through transcriptional repression of the tumor suppressor ARHI

**DOI:** 10.1186/s13058-016-0776-3

**Published:** 2016-11-21

**Authors:** Li-Liang Li, Ai-Min Xue, Bei-Xu Li, Yi-Wen Shen, Yu-Hua Li, Cheng-Liang Luo, Ming-Chang Zhang, Jie-Qing Jiang, Zu-De Xu, Jian-Hui Xie, Zi-Qin Zhao

**Affiliations:** 1 Department of Forensic Medicine, School of Basic Medical Sciences, Fudan University, 138 Yixueyuan Road, Xuhui district, Shanghai, 200032 People’s Republic of China; 2 Department of Pathology, School of Basic Medical Sciences, Fudan University, 138 Yixueyuan Road, Xuhui district, Shanghai, 200032 People’s Republic of China

In the original publication of the article [1], we noticed an error to Table 2. The numbers for each ARHI staining in the subtypes of JMJD2A were mistakenly copied from another molecular which we were simultaneously working on. This mistake went unfortunately unnoticed in the thereafter review and proofreading processes. The Table 2 shown in this erratum is the correct representation of the data from this study. The authors apologize for this error.

**Table 2:** Association between JMJD2A expression and clinicopathological parameters in 155 cases of breast cancers

	Total	JMJD2A				*P* -value	*Correlation coefficient*
		0(-) *n =* 11	1(+) *n =* 56	2(++) *n =* 53	3(+++) *n =* 35		
ERα							
Negative	52	4	18	17	13	0.778	-0.023
positive	103	7	38	36	22		
PR							
Negative	77	7	22	26	22	0.147	-0.117
positive	78	4	34	27	13		
HER2							
Negative	7	0	3	2	2	0.742	-0.027
Positive	148	11	53	51	33		
ARHI							
Negative	63	3	18	23	19	0.021*	-0.185
Positive	92	8	38	30	16		

* *p* <0.05

As a consequence, the sentence in the left column of Page 6 of 14 of the article PDF, line 4-6, should be accordingly corrected to be “Spearman correlation analysis showed that expression of JMJD2A was inversely correlated with expression of ARHI (*p* = 0.021, r = -0.185, Table 2)”.
