# Supplementary figures and images for: JMJD2A contributes to breast cancer progression through transcriptional repression of the tumor suppressor ARHI
Source: Breast Cancer Res. 2014 May 30;16(3):R56. doi: 10.1186/bcr3667 (PMC4077733; doi:10.1186/bcr3667)

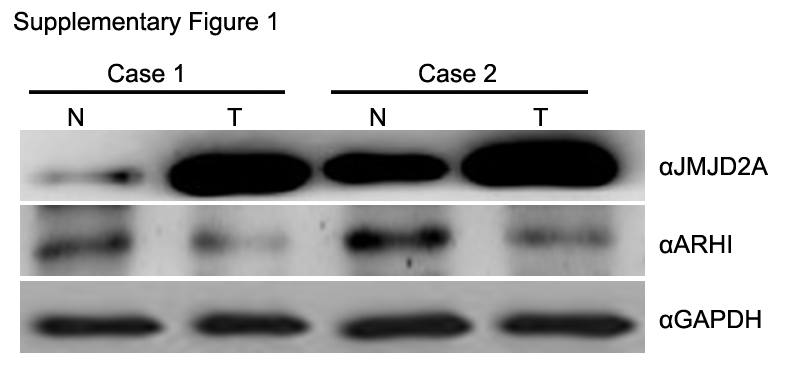

Supplement: Additional file 1: Figure S1 — JMJD2A is negatively correlated with Aplasia Ras homolog member I (ARHI) expression in breast cancer tissues. Western blot analysis of both JMJD2A and ARHI expression levels in two random human primary breast cancer (T) and paired tumor-adjacent non-cancerous breast tissues (N), with each pair taken from the same patient. [file bcr3667-S1.tiff]

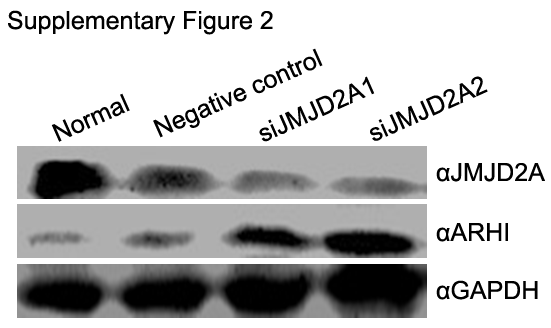

Supplement: Additional file 2: Figure S2 — Knockdown of JMJD2A could upregulate Aplasia Ras homolog member I (ARHI) expression. Two additional specific siRNAs against JMJD2A were synthesized and transfected, respectively. Negative control siRNA was also used. Both JMJD2A and ARHI expression were detected with western blot analysis. Glyceraldehyde-3-phosphate dehydrogenase (GAPDH) was used as loading control. [file bcr3667-S2.tiff]

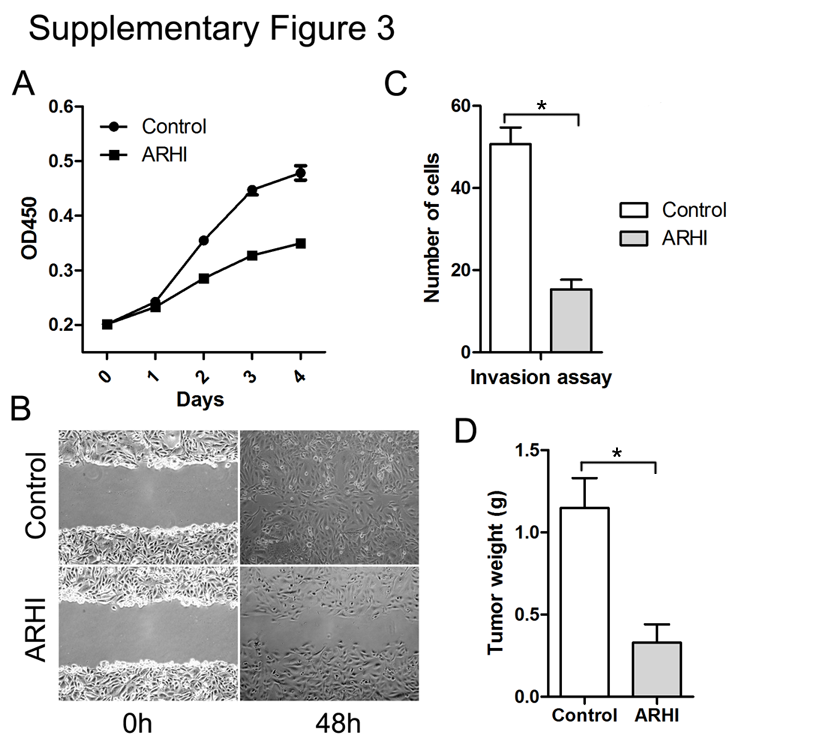

Supplement: Additional file 3: Figure S3 — Aplasia Ras homolog member I (ARHI) inhibits the tumor progression in vitro and in vivo. MDA-MB-231 cells with (ARHI) or without (Control) stable expressions of ARHI were generated using the Lenti-X system (Clontech). Analysis of cell proliferation (A), migration (wound-healing assay, B) and invasion (C) were performed. (D) The in vivo effect of ARHI was evaluated in the mouse xenograft model and the tumor weight was calculated on day 25 (n = 6 for each group). *P <0.05. [file bcr3667-S3.tiff]
